# Transcriptome-Wide *N^6^*-Methyladenosine (m^6^A) Methylome Profiling of Heat Stress in Pak-choi (*Brassica rapa* ssp. *chinensis*)

**DOI:** 10.3390/plants9091080

**Published:** 2020-08-22

**Authors:** Gaofeng Liu, Jin Wang, Xilin Hou

**Affiliations:** 1 State Key Laboratory of Crop Genetics and Germplasm Enhancement/Key Laboratory of Biology and Germplasm Enhancement of Horticultural Crops in East China, Ministry of Agriculture/Engineering Research Center of Germplasm Enhancement and Utilization of Horticultural Crops, Ministry of Education, Nanjing Agricultural University, Nanjing 210095, China; lgf@njau.edu.cn (G.L.); 2017204023@njau.edu.cn (J.W.); 2 Institute of Urban Agriculture, Chinese Academy of Agricultural Sciences, Chengdu 610213, China

**Keywords:** differentially methylated genes, heat stress, *N^6^*-methyladenosine, Pak-choi, transcriptional regulation

## Abstract

**Background:** In higher eukaryotes, *N^6^*-methyladenosine (m^6^A) is the most common internal form of messenger RNA modification. By mapping the m^6^A methyl genome in multiple species, the potential regulatory function of reversible m^6^A methylation on mRNA is revealed. Recent studies have shown that RNA m^6^A modification influences mRNA transcription, location, translation, stability, splicing, and nuclear export. However, there are not enough data on the m^6^A transcriptome-wide map and its potential biological role in the heat stress of Pak-choi (*Brassica rapa* ssp. *chinensis*). **Methods:** In this work, MeRIP-seq was used to obtain the first transcriptome-wide profiling of RNA m^6^A modification in Pak-choi. Meanwhile, the transcriptome data were obtained by analyzing the input samples’ sequencing data. **Results:** Our research indicated that with three replicates, there were 11,252 common m^6^A peaks and 9729 common m^6^A-containing genes identified in the normal (CK) and heat stress (T43) groups. It was found that m^6^A peaks were highly enriched in the 3′ untranslated region in both CK and T43 groups. About 80% of the genes have one m^6^A site. The consensus sequence of m^6^A peaks was also enriched, which showed as AAACCV (V: U/A/G). In addition, association analysis found that there is a certain relationship between the degree of m^6^A methylation and the transcription level, indicating that m^6^A plays a certain regulatory role in gene expression. **Conclusion:** This comprehensive map in the study may provide a solid basis for determining the potential function of RNA m^6^A modification in Pak-choi under normal (CK) and heat stress (T43) conditions.

## 1. Background

In higher eukaryotes, *N^6^*-methyladenosine (m^6^A) is the most common form of internal modification in long-noncoding RNAs and polyadenylated mRNAs, which was first detected in the 1970s [1]. Generally speaking, it is catalyzed by a multicomponent complex composed of two active methyltransferases (such as methyltransferase 3 and methyltransferase 14). To date, the most common of the more than 100 types of RNA modifications identified are m^6^A, m^5^C, and m^1^C RNA methylation [2,3]. Studies have shown that defects in m^6^A methylation or demethylation will lead to serious physiological consequences [4]. With continuous research in this area, it has become increasingly clear that m^6^A is essential for gene expression regulation and plays an important role in plant development.

Some previous studies have shown that RNA m^6^A plays an important role in regulating cellular metabolism and controlling the migration of embryonic stem cells in mammals [5]. In addition, RNA m^6^A can also regulate the totipotency of stem cells in mice [6] and the operation of stem cells in *Arabidopsis* [7]. In *Arabidopsis thaliana*, it was found that every 1000 nucleotides contained 0.5–0.7 sites, and each actively expressed transcript contained 0.7–1.0 sites [8,9]. In plants, m^6^A is a combination of m^6^A methyltransferase and consensus sequence RRm^6^ACH (R: G/A; H: U > A > C) [7]. In order to gain a deeper understanding of the potential biological function of RNA m^6^A modification, it is necessary to detect m^6^A modification sites within the transcriptome. With the continuous development of science and technology on m^6^A research in humans and mice, a new method has been developed to perform transcriptome-wide m^6^A localization analysis, that is, using next-generation sequencing technology (MeRIP-seq) for methylated RNA immunoprecipitation and analysis of transcriptome-wide m^6^A [10,11]. This new research method (MeRIP-seq) has also been used to obtain the first full heat-stress map in the m^6^A transcriptome range in Pak-choi. Using this method, more than two-thirds of the transcripts in *Arabidopsis* were detected with m^6^A modification [12]. Recent studies have shown that m^6^A is predominantly located near the stop codons and 3′ UTR [12]. These findings also suggest that m^6^A modification is highly dynamic and plays a specific role in regulating plant development.

Pak-choi, which is widely grown in the world today, is one of the most important vegetables in China. However, the gradual increase in ambient temperatures has affected the normal growth and development of crops, leading to a reduction in crop yield and quality [13,14]. In recent years, there have been studies on the heat stress response, including research on heat signal transduction pathways, heat stress protein identification, and transcriptional regulatory factors [15,16]. Previous studies have found that the expression level of heat stress proteins (HSPs) is affected and regulated by m^6^A on RNA [17]. Further research has found that 5′ UTR m^6^A at a single site mediates thermal stress-induced translation of HSP70 [10,18]. Currently, there is very little research on transcriptome-wide *N^6^*-methyladenosine methylome profiling of heat stress in Pak-choi and other plants. In this work, we acquire the first-ever m^6^A transcriptome-wide map of heat stress in Pak-choi. In order to further study the function of m^6^A and provide a basis for identification of m^6^A future research, we collected transcriptomes of normal (CK) and heat stress (T43) condition leaf tissues from Pak-choi. Here, we obtain the first-known m^6^A map of the transcriptome range in Pak-choi. We also compare and analyze the patterns of m^6^A distribution between CK and T43 conditions to obtain differentially methylated peaks and then analyze potential functions in gene expression regulation under high temperature stress.

## 2. Results and Discussion

### 2.1. Transcriptome-Wide Detection of m^6^A Modification in Pak-choi

**Figure 1:**
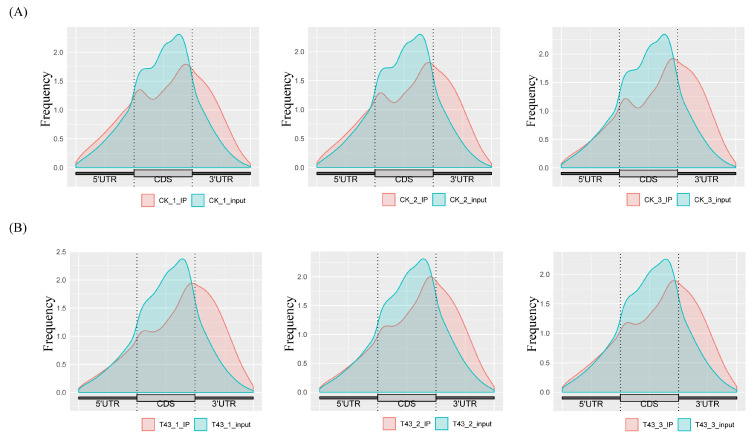
Distribution of m^6^A methylome along Pak-choi transcripts. Each transcript is divided into three parts: 5′ UTR, CDS, and 3′ UTR. (**A**) Normal (CK) group; (**B**) heat stress (T43) group.

Using Illumina Novaseq™ 6000, input and IP libraries of normal and heat stress conditions were sequenced. Statistical analysis and quality control were performed on the original data generated by RNA sequencing (Table 1). We acquired almost 80 million reads per library. After screening and quality control of the original data generated by sequencing, valid reads were mapped to the reference genome (Table 2). Among the valid reads, about 80% were uniquely mapped to the reference genome (Table 2). We next analyzed the distribution of m^6^A in the whole transcriptome for CK and T43 groups. The reads were mapped and distributed along CDS, 5′ UTR, and 3′ UTR, as depicted in Figure 1. The read frequency in 3′ UTR of m^6^A–IP samples was significantly higher than that of input samples. In Arabidopsis, studies have found that a dominant m^6^A peak near the top codon or 3′ UTR is observed in most nuclear mRNAs [11,19]. Here, in Pak-choi, our research found that m^6^A might be predominantly located in the 3′ downstream region.

**Figure 2:**
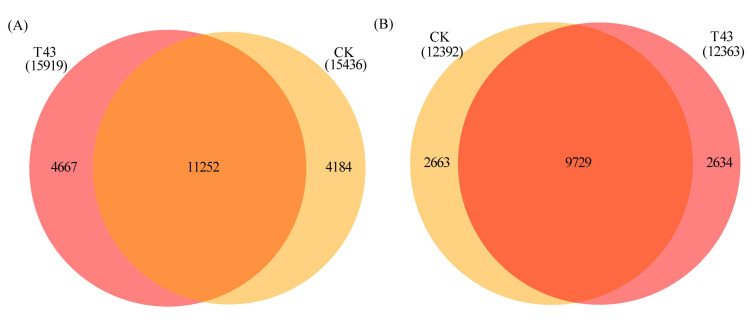
Number of overlapped m^6^A peaks (**A**) and m^6^A peak-containing genes (**B**) in CK and T43 groups.

The m^6^A peaks (actually identified as m^6^A modification sites) were identified based on a comparison of read distribution between the input and IP samples using the MeTDiff package. In the T43 and CK groups, 15,919 and 15,436 m^6^A peaks were identified, respectively (Figure 2A). The average length of peaks was 401.04 and 378.02 bp, respectively. The minimum length of peaks was 135 bp. In addition, 12,392 and 12,363 genes contained m^6^A peaks in T43 and CK groups, respectively (Figure 2B). The Veen diagram identifies 11,252 common m^6^A peaks and 9729 common m^6^A peak-containing genes (Figure 2). The GO enrichment analysis showed that the biological processes of RNA processing and methylation, salt stress response, and protein folding were significantly enriched.

### 2.2. m^6^A Topological Patterns in Pak-choi

**Figure 3:**
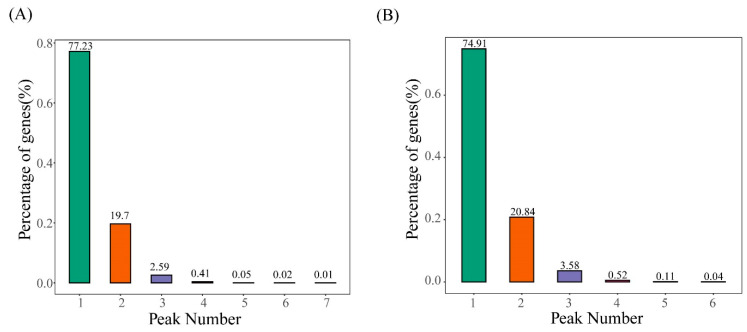
Statistics on the proportion of genes containing different numbers of m^6^A peaks. (**A**) CK group; (**B**) T43 group.

Over 90% of the methylated transcripts showed one or two m^6^A sites, whether in the CK or T43 group (Figure 3), and about 80% contained one m^6^A site, which was much higher than previously reported in *Arabidopsis* (only 27%). A previous study of m^6^A topology in *Arabidopsis* chloroplasts and mitochondria found that only one m^6^A site in these two organelles covered more than 27% of the methylated transcripts [20].

**Figure 4:**
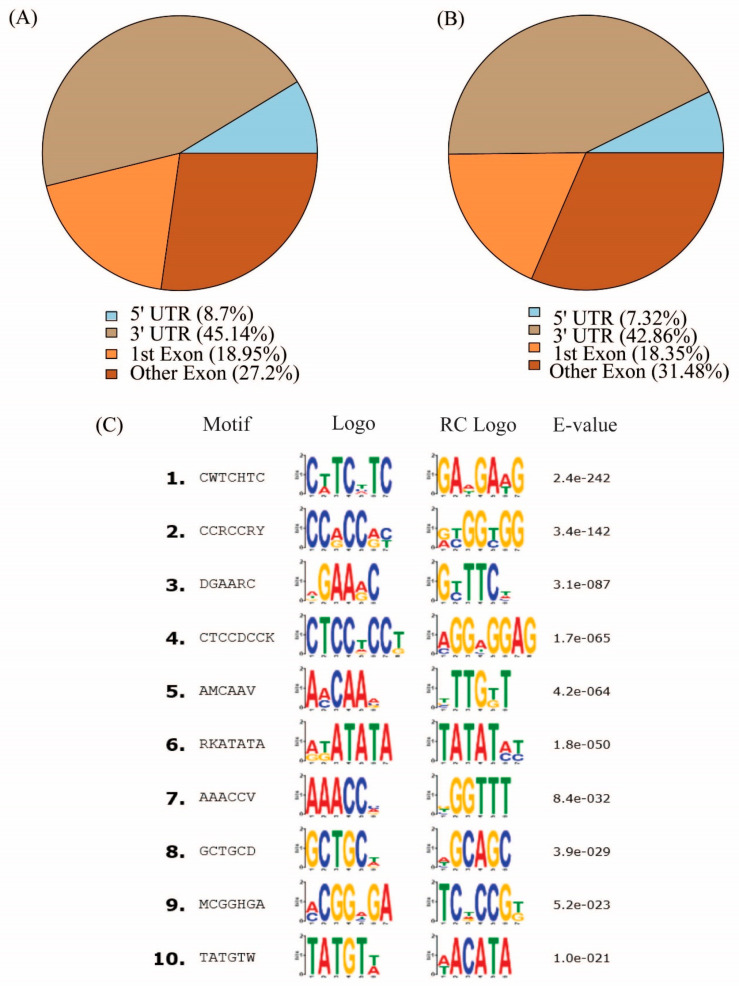
Distribution of m^6^A peaks within transcripts divided into 5′ UTR, 1st exon, 3′ UTR, and other exons. (**A**) CK group; (**B**) T43 group; (**C**) the consensus motifs enriched with m^6^A peaks in the CK group.

In order to further understand the position of m^6^A in the transcripts, the subgene profiles of the m^6^A peaks in the transcriptomes of CK and T43 groups were studied. The m^6^A peaks were assigned based on their location on different transcript segments: 5′ UTR, 3′ UTR, and exon. The results showed that the m^6^A peaks were markedly located in the 3′ untranslated region (3′ UTR; Figure 4).

**Figure 5:**
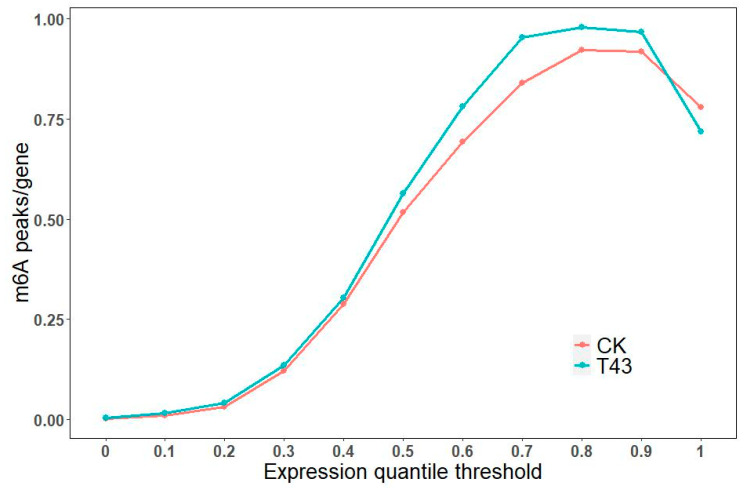
Estimation of m^6^A peaks density in Pak-choi transcripts.

To determine whether the identified m^6^A peaks share common sequence elements that are characteristic of m^6^A RNA modification, we performed an unbiased search for consensus motifs enriched in regions surrounding the m^6^A peak identified in the CK group (Figure 4C). The conserved motifs, like AAACCV (V: U/A/G; *p* = 8.4 × 10^−32^), were also enriched (Figure 4C). In order to estimate the relationships between the m^6^A peak and expression levels, the genes were equally divided into 10 groups according to expression level, from low to high, and the m^6^A density of each group was calculated separately. The m^6^A peak density increased with the increase of expression levels. We also used this information to estimate that the genes with the highest expression levels contain about 0.75 m^6^A peaks per gene in the CK and T43 groups, respectively (Figure 5).

In order to study the influence of heat stress on m^6^A modification, differential m^6^A peaks were identified between the CK and T43 groups using MeTDiff. A total of 2603 differential m^6^A peaks were identified by comparing the CK and T43 groups (Table S1). Analysis of GO enrichment of the differential m^6^A peak-containing genes showed that the biological processes of virus-induced gene silencing, freezing response, and post-translational protein modification were enriched.

### 2.3. Differentially Expressed Genes Analysis

**Figure 6:**
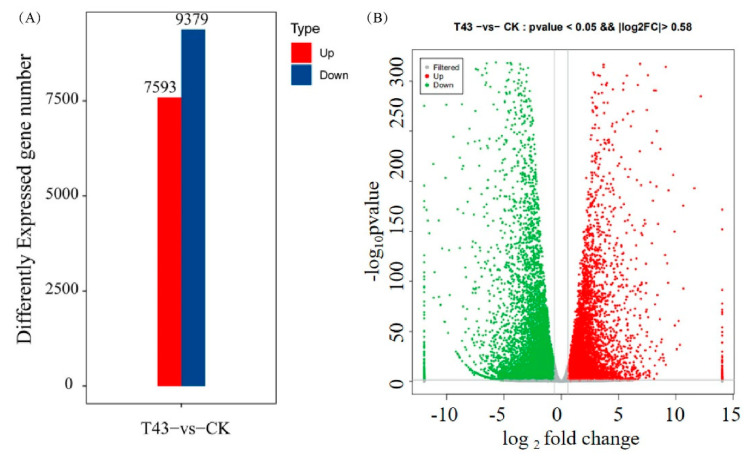
Analysis of overall results of differentially expressed genes. (**A**) Number of differentially expressed genes; (**B**) volcano analysis of differentially expressed genes.

**Figure 7:**
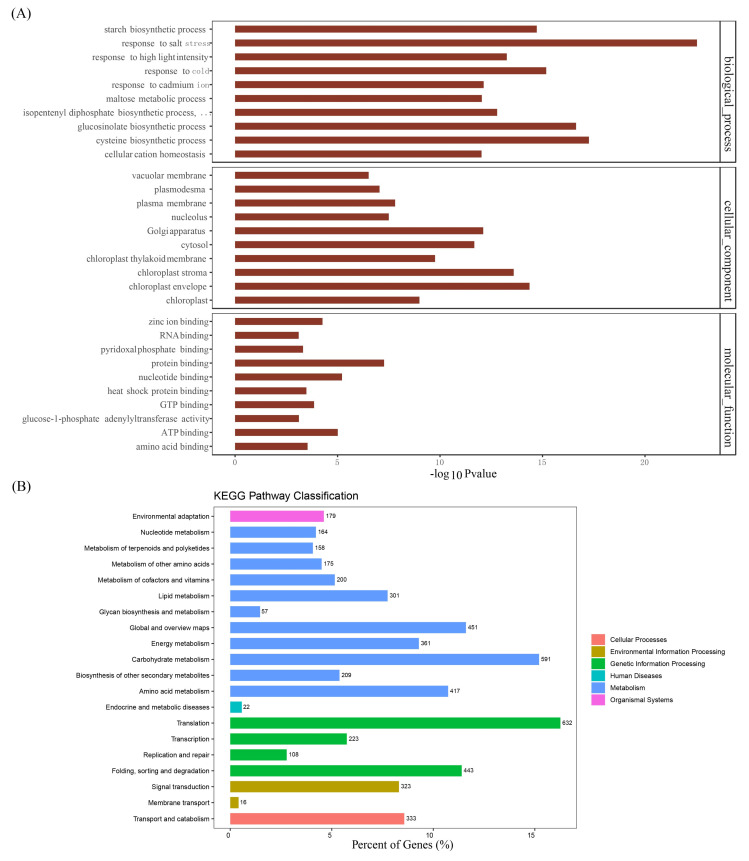
Differential expression genes enrichment analysis. (**A**) Differential expression genes GO enrichment; (**B**) differential expression genes KEGG enrichment.

To investigate the potential relationship of m^6^A and gene expression, we used FPKM (fragments per kilobase of exon model per million mapped reads) to quantify the gene expression in T43 and CK groups using the input sequencing data. As a result, 31,832 genes were detected in at least one sample (Table S2). By comparing the T43 group and the CK group, we found that 7593 genes were upregulated, while 9379 genes were downregulated, after high-temperature stress (Figure 6A). These results were further demonstrated by using gene expression density maps (Figure 6B). The differentially expressed genes (DEGs) were categorized according to GO annotations (Figure 7A). The biological processes, such as response to heat, response to cold, and response to salt stress, were enriched. Their molecular functions were primarily related to protein-binding activity. The cellular components involving the chloroplast envelope and chloroplast stroma were enriched. Through KEGG (Kyoto Encyclopedia of Genes and Genomes) enrichment analysis, we found that most differential genes were enriched in the biosynthesis of amino acids and the translation processing pathway (Figure 7B). Research findings indicated that changes in m^6^A might help regulate many genes expressed under stress [17]. In fact, Zhou et al. [21] found that m^6^A changes due to heat shock stress and activates hsp70 mRNA translation. We hypothesized that some of these differential genes might be involved in the amino acid metabolism synthesis pathway in-vivo. These findings call for further analysis of the relationship between m^6^A modification and regulation of transcriptional expression.

### 2.4. Association Analysis between Differentially Expressed Genes and Differential m^6^A Peaks

**Figure 8:**
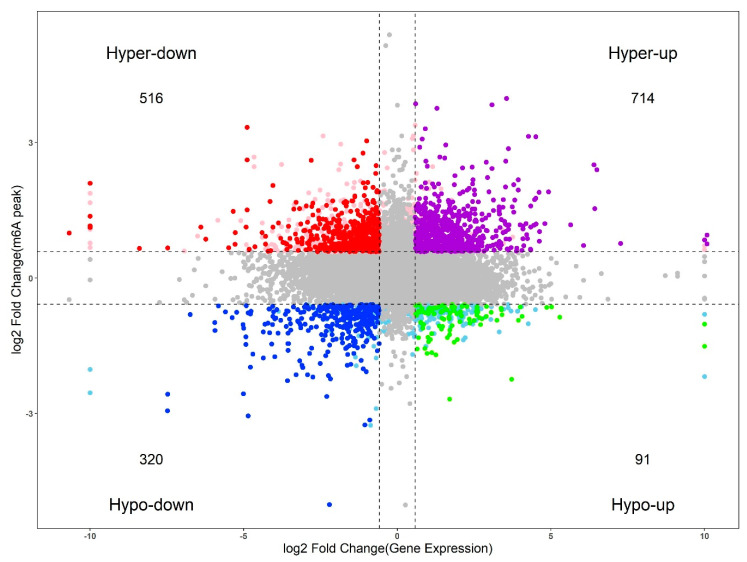
A four-image map to analyze the relationship between differential genes and differential peaks.

To further associate m^6^A modification and gene expression, codifferential gene analysis was conducted. We combined the differentially expressed genes and differential m^6^A peaks. To visually represent the link between gene expression and m^6^A methylation change, we constructed a four-image map of differential expression genes and differential m^6^A methylation genes (Figure 8). As a result, 1641 genes were identified as significant codifferential genes with the following screening criteria: fold change ≥1.5 in m^6^A peak and expression levels (Figure 8, Table S3). We found that 516 genes satisfied the significant difference in the upregulation of m^6^A and significantly downregulated their expression, and 714 differential genes satisfied the significant difference in the upregulation of m^6^A and significantly upregulated their expression. Similarly, 320 genes satisfied the significant difference in the downregulation of m^6^A and significantly downregulated their expression, and 91 genes satisfied the significant difference in the downregulation of m^6^A and significantly upregulated their expression (Figure 8). Previous studies have emphasized that m^6^A modification has a certain effect on plant development and stress resistance [22]. Further research revealed that m^6^A can inhibit site-specific transcriptome cleavage in plants, and this mechanism is necessary to properly regulate the salt-stress response transcriptome [23]. These results provide a solid foundation for further study of m^6^A modification.

## 3. Materials and Methods

### 3.1. Plant Material and Tissue Collection

An excellent variety “Suzhouqing” from Pak-choi seeds were grown in pots with a soil matrix (soil:vermiculite = 3:1) and then placed in an artificial climate room (day: 16 h at 25 °C, the light intensity was set to 150 μmol m^−2^ s^−1^; night: 8 h at 18 °C). When the seedlings reach the five-leaf stage, some seedlings were moved to the growth room without light for heat stress treatment (43 °C for 8 h); other seedlings were used as a control group (25 °C without light). Control plants and treated plants were then sampled (leaves). The fresh weight of each sample was kept at 200 mg; fresh leaves were collected, and large veins were avoided as much as possible. All leaf samples were immediately frozen in liquid nitrogen, then stored at −80 °C in a refrigerator. Three samples of each treatment were collected from all the above plants to be used in three biological replicates. At the same time, three trials were repeated.

### 3.2. Library Construction and RNA Sequencing

We used Trizol reagent (Invitrogen, Carlsbad, CA, USA) to extract total RNA from the samples obtained. The quality and quantity of the total RNA obtained were analyzed by a Bioanalyzer 2100 and an RNA 6000 Nano LabChip kit (Agilent, Palo Alto, CA, USA) with RIN value >7.0. About 200 µg of total RNA was isolated from poly (A) mRNA using magnetic beads (Invitrogen) with poly-T oligonucleotides. Then, the cleaved RNA fragment and m6A specific antibody were supplemented with BSA (0.5 μg μL^−1^). Then, we incubated those mixtures with protein A beads by elution using elution buffer. The eluted RNA was finally subjected to precipitation analysis with 75% ethanol. According to the chain-specific library prepared by the dUTP method [24], the eluted fragment containing m^6^A and the untreated input control fragment were converted into a cDNA library. The results showed that the average insertion size of the paired library was about 100 ± 50 bp. Finally, we performed 2 × 150 bp paired-end sequencing analysis on the Illumina Novaseq™ 6000 platform according to the method recommended by the supplier.

### 3.3. Data Analysis

According to the method described by our predecessors, sequence data analysis was performed on preprocessing sequencing reads and read alignments [25]. First, the original data of IP RNA-seq and input RNA-seq were analyzed by Trim Galore (version 0.3.7), adapters and low-quality (Q < 25) data were deleted, and data with readings less than 50 bp were deleted [26]. We also deleted all readings that map to multiple genomic regions, Keeping only those reads that were uniquely mapped to the reference sequence for further analysis of the m^6^A-modified peak.

### 3.4. Biological Information Analysis

To get clean data, we first used cutadapt [27] and local perl scripts to remove low-quality and contaminated sequences, followed by FastQC (http://www.bioinformatics.babraham.ac.uk/projects/fastqc/) software to clean data and conduct quality inspection and control. Finally, the default parameters of bowtie [28] were used to compare reads to the reference genome (data unpublished) to read the statistics. By using MeTDiff [29] and ChIPseeker [30], peak calling, differential m^6^A peak, and peak annotation analysis were performed, and, at the same time, by using MEME [31] and HOMER [32], motif analysis was performed on the enriched area. The gene quantification software used was StringTie [33], and the normalization mode was set to FPKM. The R package edgeR [34] was used to identify and analyze gene differences.

### 3.5. Expression and Function Analysis of Multilayer Genes

Cufflinks software (version 2.2.1) was used to measure gene expression levels per million unique mapping reads of genes per kilobase exon model input sequence reads, followed by the Cuffdiff program (version 2.2.1) to identify the number of differentially expressed genes between different samples. These genes (*p* ≤ 0.05 and normalization factor ≥1.5) are seen as differentially expressed genes [35]. GO analysis of the hypergeometric distribution test was based on the GO consortium database and R, using DAVID bioinformatics [36,37].

## 4. Conclusions

For the first time, we provide an m^6^A map on the transcriptome after heat-stress treatment in Pak-choi. More importantly, our map reveals the characteristics of m^6^A distribution in the Pak-choi transcriptome and its possible functional meaning. We also found a certain relationship between the degree of m^6^A methylation and the level of transcription, further indicating that m^6^A might play an important role in the regulation of gene expression. The construction of a comprehensive map in this study may provide a solid basis for determining the functional role of heat stress on RNA m^6^A modification in Pak-choi.

## Figures and Tables

**Table 1:** Statistics and quality control of raw data generated by sequencing.

Sample	Raw Reads	Raw Bases	Clean Reads	Clean Bases	Valid Bases	Q30	GC
CK_1_input	85.82 M	12.92 G	82.01 M	9.07 G	70.23%	87.33%	48.42%
CK_1_IP	80.33 M	12.09 G	76.98 M	10.38 G	85.82%	87.88%	47.28%
CK_2_input	86.32 M	12.99 G	83.84 M	9.49 G	73.04%	87.36%	48.52%
CK_2_IP	64.75 M	9.71 G	63.71 M	8.38 G	86.28%	89.74%	47.28%
CK_3_input	85.15 M	12.82 G	83.75 M	9.59 G	74.80%	88.50%	48.31%
CK_3_IP	73.17 M	11.01 G	69.33 M	8.84 G	80.32%	87.21%	46.96%
T43_1_input	80.40 M	12.10 G	78.73 M	8.93 G	73.79%	88.72%	48.16%
T43_1_IP	80.10 M	12.01 G	79.10 M	9.40 G	78.27%	87.65%	46.45%
T43_2_input	75.21 M	11.28 G	74.94 M	8.54 G	75.73%	89.72%	48.07%
T43_2_IP	77.01 M	11.59 G	73.52 M	9.47 G	81.67%	88.03%	46.85%
T43_3_input	85.14 M	12.82 G	81.97 M	9.50 G	74.11%	87.03%	48.03%
T43_3_IP	78.39 M	11.80 G	74.95 M	9.48 G	80.35%	88.62%	46.79%

**Table 2:** Read alignment statistics.

Sample	Total Reads	Total Mapped Reads	Multiple Mapped	Uniquely Mapped	Reads Mapped in Proper Pairs
CK_1_input	82,012,256	72,759,712 (88.72%)	4,178,829 (5.10%)	68,580,883 (83.62%)	66,920,836 (81.60%)
CK_1_IP	76,984,824	67,393,417 (87.54%)	3,465,135 (4.50%)	63,928,282 (83.04%)	59,429,546 (77.20%)
CK_2_input	83,839,082	74,908,623 (89.35%)	4,645,028 (5.54%)	70,263,595 (83.81%)	68,417,558 (81.61%)
CK_2_IP	63,714,740	56,726,613 (89.03%)	3,176,962 (4.99%)	53,549,651 (84.05%)	50,426,514 (79.14%)
CK_3_input	83,747,818	75,954,376 (90.69%)	4,321,180 (5.16%)	71,633,196 (85.53%)	69,931,644 (83.50%)
CK_3_IP	69,326,216	60,695,740 (87.55%)	3,178,979 (4.59%)	57,516,761 (82.97%)	54,734,078 (78.95%)
T43_1_input	78,733,522	71,546,508 (90.87%)	4,487,991 (5.70%)	67,058,517 (85.17%)	65,867,334 (83.66%)
T43_1_IP	79,099,558	69,007,966 (87.24%)	4,462,602 (5.64%)	64,545,364 (81.60%)	61,905,290 (78.26%)
T43_2_input	74,935,246	68,759,115 (91.76%)	4,272,256 (5.70%)	64,486,859 (86.06%)	63,435,032 (84.65%)
T43_2_IP	73,516,480	64,743,517 (88.07%)	3,957,284 (5.38%)	60,786,233 (82.68%)	58,314,510 (79.32%)
T43_3_input	81,967,046	72,968,341 (89.02%)	4,605,436 (5.62%)	68,362,905 (83.40%)	66,579,194 (81.23%)
T43_3_IP	74,954,614	66,014,828 (88.07%)	4,025,106 (5.37%)	61,989,722 (82.70%)	59,600,270 (79.52%)

## Data Availability

The RNA-seq data were deposited in the SRA database under NCBI BioProject ID PRJNA646007. All datasets supporting the manuscript conclusions are included in the article and its attached files.

## Supplementary Materials

The following are available online at https://www.mdpi.com/2223-7747/9/9/1080/s1, Table S1: T43-vs-CK-diff.p-0.05-diff.fc-1.5. Table S2: The Fpkm value of all genes in Pak-choi. Table S3: Co-differential genes in Pak-choi.

## Abbreviations

CDS	coding sequence
FPKM	fragments per kilobase of exon model per million mapped reads
GO	gene ontology
HSP	heat stress protein
KEGG	kyoto encyclopedia of genes and genomes
m^6^A	*N^6^*-methyladenosine
m^6^A-seq	RNA sequencing based on m^6^A RNA immunoprecipitation
UTR	untranslated region
